# Structure of 7-hy­droxy-3-(2-meth­oxy­phen­yl)-2-tri­fluoro­meth­yl-4*H*-chromen-4-one

**DOI:** 10.1107/S2056989017009896

**Published:** 2017-07-07

**Authors:** John Nicolson Low, Ligia R. Gomes, Alexandra Gaspar, Fernanda Borges

**Affiliations:** a Department of Chemistry, University of Aberdeen, Meston Walk, Old Aberdeen, AB24 3UE, Scotland; b FP-ENAS-Faculdade de Ciências de Saúde, Escola Superior de Saúde da UFP, Universidade Fernando Pessoa, Rua Carlos da Maia, 296, P-4200-150 Porto, Portugal; c REQUIMTE, Departamento de Química e Bioquímica, Faculdade de Ciências da Universidade do Porto, Rua do Campo Alegre, 687, P-4169-007, Porto, Portugal; d CIQ/Departamento de Quιmica e Bioquιmica, Faculdade de Ciências, Universidade do Porto, 4169-007 Porto, Portugal

**Keywords:** crystal structure, isoflavone, chromone

## Abstract

Herein, the synthesis and crystal structure of 7-hy­droxy-3-(2-meth­oxy­phen­yl)-2-tri­fluoro­meth­yl-4*H*-chromen-4-one, C_17_H_11_F_3_O_4_, are reported. This isoflavone is used as a starting material in the preparation an array of potent and competitive FPR antagonists. The pyran ring significantly deviates from planarity and the dihedral angle between the benzo­pyran mean plane and that of the exocyclic benzene ring is 88.18 (4)°. In the crystal, O—H⋯O hydrogen bonds connect the mol­ecules into *C*(8) chains propagating in the [010] direction.

## Abstract 2

The synthesis and crystal structure of 7-hy­droxy-3-(2-meth­oxy­phen­yl)-2-tri­fluoro­meth­yl-4*H*-chromen-4-one, C_17_H_11_F_3_O_4_, are reported. This isoflavone is used as a starting material in the preparation an array of potent and competitive FPR antagonists.

## Chemical context   

Isoflavones are a subclass of a larger chemical family, the flavonoids, being characterized by possessing a 3-phenyl­chromen-4-one (3-phenyl-1,4-benzopyrone) backbone instead of the 2-phenyl­chromen-4-one (3-phenyl-1,4-benzopyrone) structure of flavanones and flavones (Szeja *et al.*, 2016 ▸). Dietary isoflavones are secondary metabolites that occur in plants of the Fabaceae family and as such are present in soy beans, soy foods and legumes. The health benefits of isoflavones have been linked to cholesterol-reducing, anti-inflammatory, chemotherapeutic and anti­oxidant properties (Jie *et al.*, 2016 ▸). However, the best known property of isoflavones is related to their phytoestrogenic activity (Vitale *et al.*, 2013 ▸). More recently, isoflavones of synthetic origin have been shown to be potent and competitive antagonists of formyl peptide receptors (FPRs), playing an important role in the regulation of inflammatory processes (Schepetkin *et al.*, 2014 ▸).
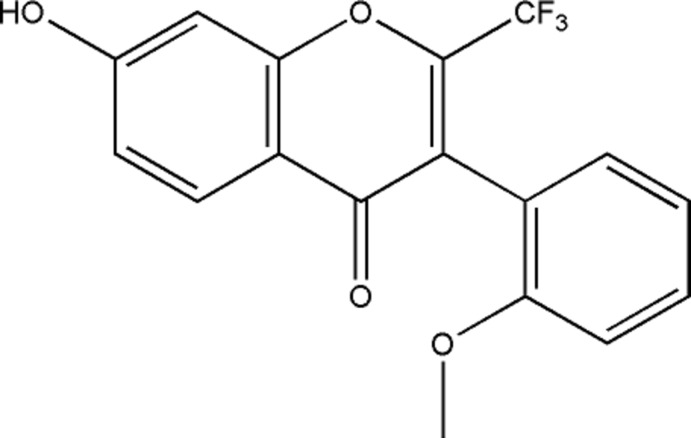


Herein we describe the synthesis and characterization of an isoflavone, 7-hy­droxy-3-(2-meth­oxy­phen­yl)-2-tri­fluoro­meth­yl-4*H*-chromen-4-one, **1**, a precursor used in the preparation of relevant FPRs antagonists.

## Structural commentary   

**Figure 1:**
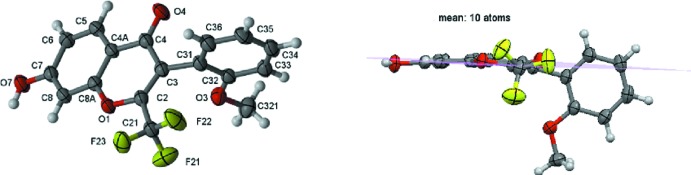
The mol­ecular structure of **1** (left) and (right)the rotation of the exocyclic benzene relative to the benzo­pyran best plane [dihedral angle = 88.18 (4)°]. Displacement ellipsoids are drawn at the 70% probability level.

The mol­ecular structure of **1** is shown in Fig. 1 ▸ (left). This compound consists of a chromone core with several substituents, *viz*. a tri­fluoro­methyl group at position 2, a 2-(meth­oxy)phenyl at position 3 and finally an hy­droxy group at position 7 of the chromone ring.

The pyran ring is not planar as the weighted average absolute torsion angle is 6.77 (7)°; for planarity this should be below 5.00° (Domenicano *et al.*, 1975 ▸). In fact, as a Cremer & Pople puckering analysis shows, the pyran ring has a twist-boat pucker with puckering amplitude *Q* = 0.1085 (13) Å, θ = 90.0 (7)° and φ = 148.6 (7)°. The fused aromatic benzo­pyran ring system shows a slight distortion from planarity as a result of the puckering of the pyran ring. The dihedral angle between the pyran ring and the exocyclic benzene ring is 89.26 (6)°. The mean plane of the ten atoms of the benzo­pyran ring system was used to evaluate the degree of twisting of the 2-meth­oxy­phenyl ring in relation to the chromone as depicted in Fig. 1 ▸ (right). The dihedral angle between the benzo­pyran mean plane and the exocyclic benzene ring is 88.18 (4)°; the major rotation is around the C3—C31 bond, which has *sp* ^3^ character, with a bond length of 1.4983 (17) Å. This conformation is to be expected and probably results from minimization of the steric hindrance between the 2-meth­oxy substituent of the exocyclic benzene ring with the voluminous –CF_3_ group and/or the oxo oxygen atom of the chromone ring.

Regarding the mean plane involving the benzo­pyran atoms, it is found that atoms O1 and C3 lie more than 0.1 Å out of it [the perpendicular vectors having values of 0.1039 (9) Å and −0.1398 (10) Å, respectively], showing again that the benzo­pyran ring itself does not show the typical planarity observed for similar chromone and coumarin structures (*e.g.* Gomes *et al.*, 2016 ▸; Reis *et al.*, 2013 ▸) in which the pyran and benzo­pyran ring systems are essentially planar. As can be seen in Table 1 ▸, the atoms of the pyran ring lie below the mean plane of the chromone benzene ring.

## Supra­molecular features   

**Figure 2:**
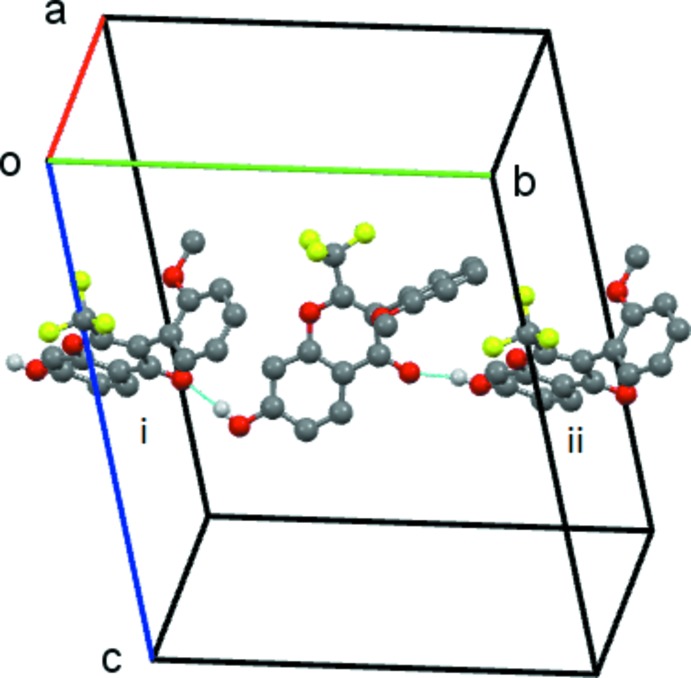
Compound **1**, the simple *C*8 chain formed by the O7—H7⋯O4^i^ hydrogen bond. This chain extends along the *b* axis. Symmetry codes: (i) −*x* + 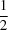, *y* − 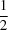, *z*; (ii) −*x* + 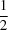, *y* + 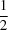, *z*). Hydrogen atoms not involved in the hydrogen bonding have been omitted.

Details of the hydrogen-bonding inter­actions are given in Table 2 ▸. The O7—H7⋯O4(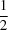 − *x*, −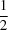 + *y*, *z*) link forms a *C*(8) chain, which runs parallel to the *b axis.* This is formed by the action of the *c*-glide plane at *y* = 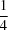, Fig. 2 ▸.

**Figure 3:**
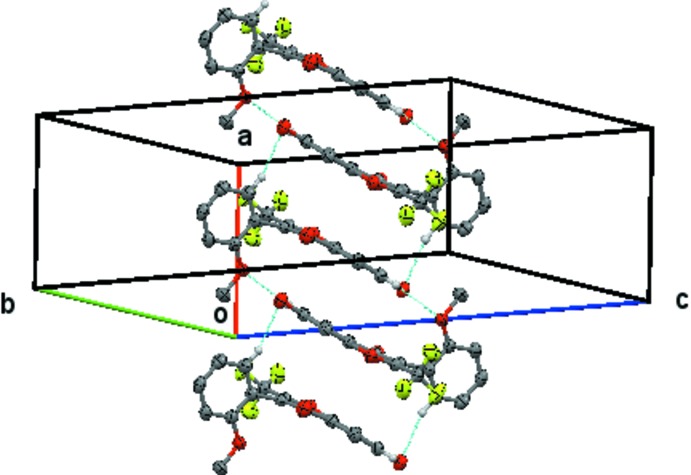
Compound **1**, view of the ladder of alternating linked 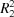
(18) and 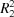
(20) structures formed by the inter­action of centrosymmetrically related pairs of C6—H6⋯O3^ii^ hydrogen bonds across the centre of symmetry at (0, 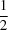, 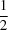) and centrosymmetrically related pairs of C36—H36⋯O7^iii^ hydrogen bonds across the centre of symmetry at (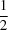, 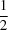, 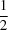). This chain extends by unit translation along the *a* axis. Hydrogen atoms not involved in the hydrogen bonding have been omitted.

The mol­ecules are linked into alternating pairs of dimers to form a ladder. The C36—H36⋯O7(1 − *x*, 1 − *y*, 1 − *z*) inter­action forms an 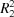
(20) centrosymmetric dimer across the centre of symmetry at (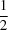, 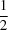, 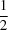). The C6—H6⋯O3(−*x*, 1 − *y*, 1 − *z*) inter­action forms an 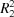
(18) centrosymmetric dimer across the centre of symmetry at (0, 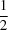, 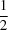). Together, these inter­actions form the ladder, which lies in plane (011) and which runs parallel to the *a* axis, Fig. 3 ▸. There are also C—H⋯π inter­actions present (Table 2 ▸).

## Hirshfeld surfaces   

The Hirshfeld surfaces and two-dimensional fingerprint (FP) plots (McKinnon *et al.*, 2004 ▸) provide complementary information concerning the inter­molecular inter­actions discussed above. They were generated using *Crystal Explorer 3.1* (Wolff *et al.*, 2012 ▸). The Hirshfeld surface mapped over *d*_norm_ is scaled between −0.250 to 1.200. The electrostatic potential (ESP) was calculated with *TONTO* (Jayatilaka & Grimwood, 2003 ▸) as implemented in *Crystal Explorer 3*.

The contributions from various contacts, listed in Table 3 ▸, were selected by the partial analysis of the FP plots. Besides the H⋯H contacts the other most significant contacts are the H⋯F/F⋯H due to the fluorine atoms on the surface. The remaining high percentage contacts are H⋯O/O⋯H that include the relevant C—H⋯O and the O—H⋯O inter­molecular inter­actions and also the H⋯C/C⋯H contacts including C—H⋯C contacts. The percentage of C⋯C contacts is 6.1% but they are too long to be considered as π–π stacking. The structure has four oxygen atoms, defining different functional groups, that may act as acceptors for hydrogen bonds: one oxo group, a meth­oxy group, a hydroxyl group and an alk­oxy O atom, all of which participate in short atom–atom contacts with the exception of the chromone alk­oxy O atom.

**Figure 4:**
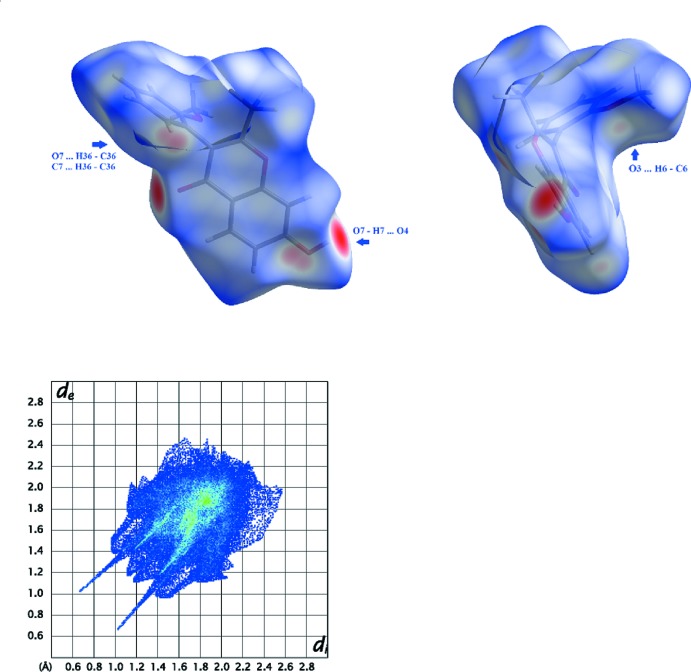
Views of the Hirshfeld surface mapped over *d*_norm_ for **1** and the corresponding FP plot. The highlighted red spots with large area on the top left image indicate O⋯H contact points involving the carbonyl oxygen atom of the chromone core and the hydrogen atom of the hydroxyl substituent while the pair of superposed light-red spots indicate C⋯H and O⋯H close contacts. The small red-spot areas on the concave and convex face of the right image are due to C⋯H close contacts. The bottom of the figure presents the FP plot for mol­ecule **1** The light-blue area in the middle of the FP plot at *d*_e_/*d*_i_ ∼ 1.9 Å shows a higher frequency of the pixels that are due to C⋯C contacts. The sharp spikes pointing to southwest are due to O⋯H contacts: the inner one on the right is related to O⋯H contacts and the short wings due to C⋯H close contacts (see Fig. 5 ▸ for details).

**Figure 5:**
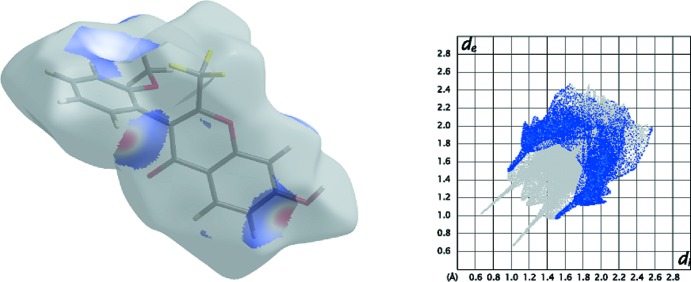
Detail of the FP plot for **1** highlighting the H⋯C contacts (blue) and the corresponding areas in the Hirshfeld surface; The blue area covers the C⋯H/ H⋯C close contacts and displays two small wings as well as a pair of short spikes pointing to southwest ending at (*d*_e/_ *d*_i_)/(1.5/1.0) Å and *vice versa* that reflect the C36—H36⋯C7 contact area.

The Hirshfeld surfaces mapped over *d*_norm_ for **1** (see Fig. 4 ▸) show three sets of complementary red spot areas: one of those pairs consist of two intense red areas, circular in shape, that are located near the carbonyl oxygen atom O4 and near the hydroxyl substituent. This close contact accounts for the O4⋯H7—O7 hydrogen bond as indicated in Fig. 4 ▸. Another pair is consists of two light-red areas resulting from the overlap of two red spots near H36 and near the hydroxyl group; they suggest inter­actions between this hydrogen atom and O7 (that forms a C36—H36⋯O7 hydrogen contact) and with the carbon atom C7 of the chromone ring (Fig. 5 ▸ contains a detail of this contact and the corresponding FP plot area). Finally, there are two complementary very light-red spots of small diameter that suggest the existence of a close contact involving the oxygen atom O3 of the meth­oxy substituent with hydrogen atom H8 of the chromone ring (O3⋯H6—C6).

The FP plot for **1** is included in Fig. 4 ▸. The light-blue area in the middle of it at *d*_e_/*d*_i_ approximately equal to 1.9 Å shows a higher frequency of the pixels that are due to C⋯C contacts. The sharp spikes pointing to the southwest are due to O⋯H contacts and the short wings due to C⋯H close contacts (see Fig. 5 ▸ for details).

**Figure 6:**
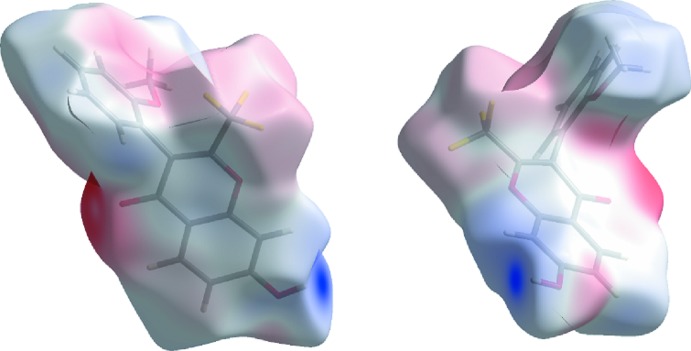
The electrostatic potential surfaces for **1** (ranging from −0.0920 to 0.2582 a.u.). The surfaces show the complementary of electropositive area (blue) near the hydrogen atom of the hydroxyl substituent and red electronegative area surrounding the vicinity of the lone pairs of the oxo oxygen atom O4.

In Fig. 6 ▸ the mapping of the mol­ecular electrostatic potential (ESP) in the context of crystal packing is shown. As the Hirshfeld surface partitions of the crystal space give non-overlapping volumes associated with each mol­ecule, these surfaces give a kind of ‘electrostatic complementarity’; red areas indicate negative electrostatic potential while blue areas indicate a positive one. The ESP mapped in the Hirshfeld surface for **1** reveals a red area of strongly negative electrostatic potential surrounding the carbonyl region of the chromone and light red areas surrounding the fluorine atoms of the –CF_3_ and as well on the areas covering the oxygen atoms of the hydroxyl and meth­oxy substituents showing the negative electrostatic potential. The blue region, strongly electropositive, is predominantly located on the hydrogen atom of the hydroxyl substituent and the light electropositive blue patch areas are also surrounding the H atoms of the meth­oxy substituent and well as H8 and H6 hydrogen atoms of the chromone. The remainder of the Hirshfeld surface is close to neutrality as seen by the grey regions. Thus, the figures highlight the electrostatic complementarity in the O4⋯H7—O7 contact as well as in the O3⋯H6—C6 contact.

## Database survey   

A search made in the Cambridge Structural Database, (Groom *et al.*, 2016 ▸), revealed the existence of seven polymorphic and pseudopolymorphic crystal structures of 7-hy­droxy-3-phenyl-4*H*-chromen-4-one (Gong *et al.*, 2016 ▸). In these structures, the pyran rings are essentially planar. The dihedral angles between the benzo­pyran ten-membered ring mean plane and the exocyclic benzene ring are given below. KUZJEW, 7-hy­droxy-3-phenyl-4*H*-chromen-4-one: 2-methyl­propan-2-ol solvate, dihedral angle= 48.28 (7)°. KUZJIA, 7-hy­droxy-3-phenyl-4*H*-chromen-4-one, dihedral angle = 55.23 (8)°. KUZJIA01, 7-hy­droxy-3-phenyl-4*H*-chromen-4-one, dihedral angle = 56.83 (7)°(mol­ecule *A*), 48.27 (6)° (mol­ecule *B*). KUZNIE, 7-hy­droxy-3-phenyl-4*H*-chromen-4-one; dimethyl sulfoxide solvate, dihedral angle = 45.91 (7)°. KUZJUM, 7-hy­droxy-3-phenyl-4*H*-chromen-4-one *N*,*N*-di­methyl­formamide solvate, dihedral angle = 41.70 (7)°. KUZKAT, 7-hy­droxy-3-phenyl-4*H*-chromen-4-one propan-1-ol, solvate, dihedral angle = 45.18 (9)°. KUZKEX, 7-hy­droxy-3-phenyl-4*H*-chromen-4-one butan-1-ol solvate, dihedral angle = 45.11 (11)°. In all solvated structures, the 7-OH hydroxyl group is involved in hydrogen bonding with the solvent. In the two KUZJIA(01) structures, –OH⋯O chains are formed as in **1**.

## Synthesis and crystallization   

The title compound was obtained by a two-step synthesis (Balasubramanian & Nair, 2000 ▸; Eiffe *et al.*, 2009 ▸). Resorcinol and 2-meth­oxy­phenyl­acetic acid, in equimolar amounts, were suspended in boron trifluoride diethyl etherate (BF_3_·Et_2_O) and heated at 358 K, for 90 min. Then the mixture was poured into water and stirred until the formation of a solid and extracted with ethyl acetate. The combined organic phases were washed with water, dried over anhydrous sodium sulfate, filtered and evaporated. The product was recrystallized from ethyl acetate solution and used in the subsequent reaction to obtain the isoflavone.

1-(2,4-Di­hydroxy­phen­yl)-2-(2-meth­oxy­phen­yl)ethan-1-one (1 mmol), tri­fluoro­acetic anhydride (3 mmol) and tri­methyl­amine (2 ml) were refluxed for 1 h. After cooling, water (15 ml) was added. The solution was acidified (pH 5) with 2 *M* HCl and stirred at room temperature for 2 h. After extraction with ethyl acetate, the combined organic phases were washed with water, dried over anhydrous sodium sulfate, filtered and evaporated. The isoflavone was recrystallized from ethyl acetate solution. Overall yield: 55%

^1^H NMR (DMSO-*d*_6_): 3.69 (1H, *s*), 6.95 (1H, *d, J* = 2.20 Hz), 7.00 (1H, *ddd, J* = 0.97, 7.45, 7.45 Hz), 7.02 (1H, *dd, J* = 2.26, 8.82 Hz), 7.09 (1H, *dd, J* = 0.90, 8.38 Hz), 7.15 (1H, *dd, J* = 1.70, 7.46 Hz), 7.42 (1H, *ddd, J* = 1.73, 7.47, 8.31 Hz), 7.92 (1H, *d, J* = 8.76 Hz), 11.13 (1H, *s*).

## Refinement   

Crystal data, data collection and structure refinement details are summarized in Table 4 ▸. The hydroxyl H atom, H7, was refined isotropically. All other H atoms were treated as riding atoms: C—H = 0.95–0.98 Å with *U*_iso_ = 1.5*U*_eq_(C-methyl) and 1.2*U*_eq_(C) for other H atoms.

## Supplementary Material

Crystal structure: contains datablock(s) I, global. DOI: 10.1107/S2056989017009896/hb7688sup1.cif

Structure factors: contains datablock(s) I. DOI: 10.1107/S2056989017009896/hb7688Isup2.hkl

Supporting information file. DOI: 10.1107/S2056989017009896/hb7688Isup3.cml

CCDC reference: 1453086

Additional supporting information:  crystallographic information; 3D view; checkCIF report

## Figures and Tables

**Table 1:** Deviations (in Å) of the pyran ring atoms and attached atoms from the mean plane of the chromone benzene ring

Atom	O1	C2	C3′	C4	C21	C31	O4
Distance	−0.0092 (18)	−0.243 (2)	−0.380 (2)	−0.155 (2)	−0.372 (3)	−0.805 (3)	−0.1267 (16)

**Table 2:** Hydrogen-bond geometry (Å, °)

*D*—H⋯*A*	*D*—H	H⋯*A*	*D*⋯*A*	*D*—H⋯*A*
O7—H7⋯O4^i^	0.87 (2)	1.79 (2)	2.6416 (13)	166 (2)
C6—H6⋯O3^ii^	0.95	2.59	3.4309 (16)	148
C36—H36⋯O7^iii^	0.95	2.49	3.3886 (18)	157
C8—H8⋯*Cg*3^iv^	0.95	2.93	3.5972 (14)	128
C321—H32b⋯*Cg*3^v^	0.98	2.75	3.6348 (17)	150

Symmetry codes: (i) 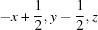; (ii) 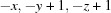; (iii) 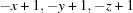; (iv) 
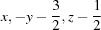
; (v) 
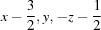
.

**Table 3:** Percentages for the most relevant atom–atom contacts in **1**

H⋯H’	H⋯O/O⋯H	H⋯F/F⋯H	H⋯C/C⋯H	C⋯C	C⋯O/O⋯C	O⋯F/F⋯C	O⋯O	F⋯O/O⋯F	F⋯F
22.1	18.3	25.1	18.3	6.1	2.3	0.2	3.0	0.8	3.4

**Table 4:** Experimental details

Crystal data	
Chemical formula	C_17_H_11_F_3_O_4_
*M*_r_	336.27
Crystal system, space group	Orthorhombic, *P* *b* *c* *a*
Temperature (K)	100
*a*, *b*, *c* (Å)	7.9147 (5), 16.2171 (11), 22.5254 (16)
*V* (Å^3^)	2891.2 (3)
*Z*	8
Radiation type	Mo *K*α
μ (mm^−1^)	0.14
Crystal size (mm)	0.21 × 0.07 × 0.01

Data collection	
Diffractometer	Rigaku AFC12
Absorption correction	Multi-scan (*CrystalClear-SM Expert*; Rigaku, 2012 ▸)
*T*_min_, *T*_max_	0.775, 1.000
No. of measured, independent and observed [*I* > 2σ(*I*)] reflections	35487, 3314, 2777
*R*_int_	0.058
(sin θ/λ)_max_ (Å^−1^)	0.649

Refinement	
*R*[*F* ^2^ > 2σ(*F* ^2^)], *wR*(*F* ^2^), *S*	0.036, 0.103, 1.02
No. of reflections	3314
No. of parameters	222
H-atom treatment	H atoms treated by a mixture of independent and constrained refinement
Δρ_max_, Δρ_min_ (e Å^−3^)	0.34, −0.21

Computer programs: *CrystalClear-SM Expert* (Rigaku, 2012 ▸), *SHELXT* (Sheldrick, 2015*a* ▸), *OSCAIL* (McArdle *et al.*, 2004 ▸), *ShelXle* (Hübschle *et al.*, 2011 ▸), *SHELXL2014* (Sheldrick, 2015*b* ▸), *Mercury* (Macrae *et al.*, 2006 ▸) and *PLATON* (Spek, 2009 ▸).

## supplementary crystallographic information

### Crystal data

**Table:**

C_17_H_11_F_3_O_4_	*D*_x_ = 1.545 Mg m^−^^3^
*M**_r_* = 336.27	Mo *K*α radiation, λ = 0.71075 Å
Orthorhombic, *P**b**c**a*	Cell parameters from 31907 reflections
*a* = 7.9147 (5) Å	θ = 2.5–27.5°
*b* = 16.2171 (11) Å	µ = 0.14 mm^−^^1^
*c* = 22.5254 (16) Å	*T* = 100 K
*V* = 2891.2 (3) Å^3^	Plate, colourless
*Z* = 8	0.21 × 0.07 × 0.01 mm
*F*(000) = 1376	

### Data collection

**Table:**

Rigaku AFC12 diffractometer	3314 independent reflections
Radiation source: Rotating Anode	2777 reflections with *I* > 2σ(*I*)
Detector resolution: 28.5714 pixels mm^-1^	*R*_int_ = 0.058
profile data from ω–scans	θ_max_ = 27.5°, θ_min_ = 2.5°
Absorption correction: multi-scan (*CrystalClear-SM Expert*; Rigaku, 2012)	*h* = −10→10
*T*_min_ = 0.775, *T*_max_ = 1.000	*k* = −20→20
35487 measured reflections	*l* = −29→29

### Refinement

**Table:**

Refinement on *F*^2^	0 restraints
Least-squares matrix: full	Hydrogen site location: mixed
*R*[*F*^2^ > 2σ(*F*^2^)] = 0.036	H atoms treated by a mixture of independent and constrained refinement
*wR*(*F*^2^) = 0.103	*w* = 1/[σ^2^(*F*_o_^2^) + (0.0543*P*)^2^ + 1.0823*P*] where *P* = (*F*_o_^2^ + 2*F*_c_^2^)/3
*S* = 1.02	(Δ/σ)_max_ < 0.001
3314 reflections	Δρ_max_ = 0.34 e Å^−^^3^
222 parameters	Δρ_min_ = −0.21 e Å^−^^3^

### Special details

**Table:**

Geometry. All esds (except the esd in the dihedral angle between two l.s. planes) are estimated using the full covariance matrix. The cell esds are taken into account individually in the estimation of esds in distances, angles and torsion angles; correlations between esds in cell parameters are only used when they are defined by crystal symmetry. An approximate (isotropic) treatment of cell esds is used for estimating esds involving l.s. planes.

### Fractional atomic coordinates and isotropic or equivalent isotropic displacement parameters (Å^2^)

**Table:**

	*x*	*y*	*z*	*U*_iso_*/*U*_eq_	
F21	0.37922 (12)	0.49225 (7)	0.27664 (4)	0.0476 (3)	
F22	0.59157 (13)	0.56872 (5)	0.29861 (4)	0.0459 (3)	
F23	0.59737 (12)	0.43950 (5)	0.31761 (4)	0.0416 (2)	
O1	0.38627 (12)	0.44652 (5)	0.40256 (4)	0.0248 (2)	
O3	0.13208 (12)	0.65679 (5)	0.33431 (4)	0.0276 (2)	
O4	0.29964 (14)	0.66084 (5)	0.49162 (4)	0.0323 (2)	
O7	0.08054 (13)	0.29227 (6)	0.54664 (4)	0.0293 (2)	
H7	0.114 (3)	0.2531 (13)	0.5233 (9)	0.055 (6)*	
C2	0.42085 (16)	0.52119 (7)	0.37796 (6)	0.0235 (3)	
C3	0.38708 (16)	0.59517 (7)	0.40248 (6)	0.0232 (3)	
C4	0.31834 (16)	0.59616 (7)	0.46352 (6)	0.0243 (3)	
C5	0.17944 (17)	0.50964 (7)	0.54149 (6)	0.0253 (3)	
H5	0.1607	0.5575	0.5649	0.030*	
C4A	0.26922 (16)	0.51638 (7)	0.48767 (5)	0.0230 (3)	
C6	0.11873 (17)	0.43488 (7)	0.56045 (6)	0.0256 (3)	
H6	0.0579	0.4311	0.5967	0.031*	
C7	0.14709 (17)	0.36333 (7)	0.52579 (6)	0.0248 (3)	
C8	0.24070 (17)	0.36720 (7)	0.47361 (6)	0.0244 (3)	
H8	0.2647	0.3190	0.4512	0.029*	
C8A	0.29805 (16)	0.44429 (7)	0.45536 (5)	0.0224 (3)	
C21	0.49789 (19)	0.50614 (7)	0.31732 (6)	0.0277 (3)	
C31	0.40573 (17)	0.67677 (7)	0.37198 (6)	0.0244 (3)	
C32	0.26803 (16)	0.70816 (7)	0.33969 (5)	0.0237 (3)	
C33	0.27695 (18)	0.78732 (8)	0.31499 (6)	0.0274 (3)	
H33	0.1837	0.8090	0.2935	0.033*	
C34	0.42290 (19)	0.83407 (8)	0.32209 (6)	0.0303 (3)	
H34	0.4285	0.8878	0.3055	0.036*	
C35	0.56005 (19)	0.80327 (8)	0.35305 (6)	0.0314 (3)	
H35	0.6600	0.8352	0.3571	0.038*	
C36	0.55002 (18)	0.72477 (8)	0.37832 (6)	0.0290 (3)	
H36	0.6433	0.7039	0.4002	0.035*	
C321	−0.01472 (19)	0.69009 (9)	0.30555 (7)	0.0343 (3)	
H32A	−0.1047	0.6486	0.3052	0.051*	
H32B	0.0137	0.7053	0.2647	0.051*	
H32C	−0.0533	0.7391	0.3271	0.051*	

### Atomic displacement parameters (Å^2^)

**Table:**

	*U*^11^	*U*^22^	*U*^33^	*U*^12^	*U*^13^	*U*^23^
F21	0.0446 (6)	0.0703 (7)	0.0280 (5)	0.0018 (5)	−0.0036 (4)	−0.0067 (4)
F22	0.0623 (6)	0.0276 (4)	0.0478 (5)	−0.0090 (4)	0.0258 (5)	−0.0027 (4)
F23	0.0549 (6)	0.0325 (4)	0.0373 (5)	0.0188 (4)	0.0129 (4)	0.0034 (3)
O1	0.0320 (5)	0.0160 (4)	0.0265 (5)	0.0010 (3)	0.0023 (4)	−0.0003 (3)
O3	0.0260 (5)	0.0259 (4)	0.0308 (5)	0.0002 (4)	−0.0032 (4)	0.0043 (4)
O4	0.0432 (6)	0.0178 (4)	0.0357 (5)	−0.0001 (4)	0.0037 (4)	−0.0044 (4)
O7	0.0375 (6)	0.0178 (4)	0.0327 (5)	−0.0016 (4)	0.0040 (4)	0.0008 (4)
C2	0.0248 (6)	0.0188 (6)	0.0269 (6)	0.0001 (5)	−0.0016 (5)	0.0017 (4)
C3	0.0228 (6)	0.0189 (5)	0.0280 (6)	0.0008 (4)	−0.0030 (5)	0.0005 (5)
C4	0.0251 (6)	0.0184 (5)	0.0294 (6)	0.0017 (5)	−0.0031 (5)	−0.0006 (5)
C5	0.0307 (7)	0.0191 (5)	0.0262 (6)	0.0022 (5)	−0.0019 (5)	−0.0027 (5)
C4A	0.0257 (6)	0.0174 (5)	0.0258 (6)	0.0016 (5)	−0.0030 (5)	−0.0012 (4)
C6	0.0299 (7)	0.0223 (6)	0.0245 (6)	0.0027 (5)	0.0006 (5)	−0.0003 (5)
C7	0.0270 (7)	0.0188 (5)	0.0286 (6)	0.0008 (5)	−0.0037 (5)	0.0018 (5)
C8	0.0296 (7)	0.0161 (5)	0.0275 (6)	0.0012 (5)	−0.0022 (5)	−0.0018 (4)
C8A	0.0246 (6)	0.0197 (6)	0.0231 (6)	0.0016 (5)	−0.0017 (5)	−0.0007 (4)
C21	0.0336 (7)	0.0202 (6)	0.0294 (6)	0.0015 (5)	0.0020 (5)	0.0004 (5)
C31	0.0296 (7)	0.0176 (5)	0.0258 (6)	0.0016 (5)	0.0017 (5)	0.0001 (4)
C32	0.0276 (6)	0.0203 (5)	0.0231 (6)	0.0016 (5)	0.0025 (5)	−0.0006 (4)
C33	0.0339 (7)	0.0223 (6)	0.0259 (6)	0.0055 (5)	0.0028 (5)	0.0019 (5)
C34	0.0426 (8)	0.0196 (6)	0.0287 (7)	0.0007 (5)	0.0081 (6)	0.0019 (5)
C35	0.0361 (7)	0.0220 (6)	0.0361 (7)	−0.0056 (5)	0.0043 (6)	−0.0028 (5)
C36	0.0301 (7)	0.0222 (6)	0.0346 (7)	0.0000 (5)	−0.0015 (5)	−0.0007 (5)
C321	0.0292 (7)	0.0375 (7)	0.0362 (7)	0.0034 (6)	−0.0058 (6)	0.0056 (6)

### Geometric parameters (Å, º)

**Table:**

F21—C21	1.3314 (17)	C6—C7	1.4164 (17)
F22—C21	1.3256 (15)	C6—H6	0.9500
F23—C21	1.3371 (15)	C7—C8	1.3908 (19)
O1—C2	1.3594 (15)	C8—C8A	1.3921 (17)
O1—C8A	1.3796 (15)	C8—H8	0.9500
O3—C32	1.3662 (15)	C31—C36	1.3895 (19)
O3—C321	1.4356 (16)	C31—C32	1.4057 (18)
O4—C4	1.2340 (15)	C32—C33	1.4008 (17)
O7—C7	1.3514 (15)	C33—C34	1.391 (2)
O7—H7	0.87 (2)	C33—H33	0.9500
C2—C3	1.3475 (17)	C34—C35	1.384 (2)
C2—C21	1.5158 (18)	C34—H34	0.9500
C3—C4	1.4788 (18)	C35—C36	1.3967 (18)
C3—C31	1.4983 (17)	C35—H35	0.9500
C4—C4A	1.4564 (16)	C36—H36	0.9500
C5—C6	1.3723 (17)	C321—H32A	0.9800
C5—C4A	1.4094 (18)	C321—H32B	0.9800
C5—H5	0.9500	C321—H32C	0.9800
C4A—C8A	1.3960 (16)		
			
C2—O1—C8A	118.46 (9)	F22—C21—F21	107.78 (11)
C32—O3—C321	116.62 (10)	F22—C21—F23	106.92 (12)
C7—O7—H7	107.1 (13)	F21—C21—F23	106.39 (11)
C3—C2—O1	125.88 (12)	F22—C21—C2	112.84 (10)
C3—C2—C21	126.33 (11)	F21—C21—C2	111.33 (12)
O1—C2—C21	107.75 (10)	F23—C21—C2	111.26 (10)
C2—C3—C4	117.63 (11)	C36—C31—C32	119.18 (11)
C2—C3—C31	125.38 (12)	C36—C31—C3	121.91 (12)
C4—C3—C31	116.93 (10)	C32—C31—C3	118.73 (11)
O4—C4—C4A	122.10 (12)	O3—C32—C33	124.28 (12)
O4—C4—C3	122.03 (11)	O3—C32—C31	115.83 (10)
C4A—C4—C3	115.84 (10)	C33—C32—C31	119.89 (12)
C6—C5—C4A	120.84 (11)	C34—C33—C32	119.71 (13)
C6—C5—H5	119.6	C34—C33—H33	120.1
C4A—C5—H5	119.6	C32—C33—H33	120.1
C8A—C4A—C5	117.78 (11)	C35—C34—C33	120.85 (12)
C8A—C4A—C4	120.36 (11)	C35—C34—H34	119.6
C5—C4A—C4	121.66 (11)	C33—C34—H34	119.6
C5—C6—C7	119.78 (12)	C34—C35—C36	119.34 (13)
C5—C6—H6	120.1	C34—C35—H35	120.3
C7—C6—H6	120.1	C36—C35—H35	120.3
O7—C7—C8	122.67 (11)	C31—C36—C35	121.02 (13)
O7—C7—C6	116.44 (12)	C31—C36—H36	119.5
C8—C7—C6	120.88 (11)	C35—C36—H36	119.5
C7—C8—C8A	117.63 (11)	O3—C321—H32A	109.5
C7—C8—H8	121.2	O3—C321—H32B	109.5
C8A—C8—H8	121.2	H32A—C321—H32B	109.5
O1—C8A—C8	116.31 (10)	O3—C321—H32C	109.5
O1—C8A—C4A	120.69 (10)	H32A—C321—H32C	109.5
C8—C8A—C4A	123.01 (12)	H32B—C321—H32C	109.5
			
C8A—O1—C2—C3	4.95 (19)	C4—C4A—C8A—O1	5.63 (18)
C8A—O1—C2—C21	−172.99 (10)	C5—C4A—C8A—C8	0.76 (19)
O1—C2—C3—C4	5.0 (2)	C4—C4A—C8A—C8	−174.13 (12)
C21—C2—C3—C4	−177.39 (12)	C3—C2—C21—F22	25.4 (2)
O1—C2—C3—C31	−171.91 (12)	O1—C2—C21—F22	−156.64 (11)
C21—C2—C3—C31	5.7 (2)	C3—C2—C21—F21	−95.91 (15)
C2—C3—C4—O4	172.60 (12)	O1—C2—C21—F21	82.03 (13)
C31—C3—C4—O4	−10.19 (19)	C3—C2—C21—F23	145.61 (13)
C2—C3—C4—C4A	−9.24 (17)	O1—C2—C21—F23	−36.46 (15)
C31—C3—C4—C4A	167.97 (11)	C2—C3—C31—C36	−95.96 (16)
C6—C5—C4A—C8A	−1.73 (19)	C4—C3—C31—C36	87.07 (16)
C6—C5—C4A—C4	173.08 (12)	C2—C3—C31—C32	88.86 (16)
O4—C4—C4A—C8A	−177.67 (12)	C4—C3—C31—C32	−88.10 (14)
C3—C4—C4A—C8A	4.17 (18)	C321—O3—C32—C33	−5.40 (18)
O4—C4—C4A—C5	7.6 (2)	C321—O3—C32—C31	175.24 (11)
C3—C4—C4A—C5	−170.51 (12)	C36—C31—C32—O3	178.68 (11)
C4A—C5—C6—C7	0.3 (2)	C3—C31—C32—O3	−6.01 (17)
C5—C6—C7—O7	−178.66 (12)	C36—C31—C32—C33	−0.71 (18)
C5—C6—C7—C8	2.2 (2)	C3—C31—C32—C33	174.60 (11)
O7—C7—C8—C8A	177.81 (12)	O3—C32—C33—C34	−178.71 (12)
C6—C7—C8—C8A	−3.06 (19)	C31—C32—C33—C34	0.63 (18)
C2—O1—C8A—C8	169.42 (11)	C32—C33—C34—C35	0.3 (2)
C2—O1—C8A—C4A	−10.35 (17)	C33—C34—C35—C36	−1.2 (2)
C7—C8—C8A—O1	−178.16 (11)	C32—C31—C36—C35	−0.2 (2)
C7—C8—C8A—C4A	1.6 (2)	C3—C31—C36—C35	−175.30 (12)
C5—C4A—C8A—O1	−179.48 (11)	C34—C35—C36—C31	1.1 (2)

### Hydrogen-bond geometry (Å, º)

**Table:**

*D*—H···*A*	*D*—H	H···*A*	*D*···*A*	*D*—H···*A*
O7—H7···O4^i^	0.87 (2)	1.79 (2)	2.6416 (13)	166 (2)
C6—H6···O3^ii^	0.95	2.59	3.4309 (16)	148
C36—H36···O7^iii^	0.95	2.49	3.3886 (18)	157
C8—H8···*Cg*3^iv^	0.95	2.93	3.5972 (14)	128
C321—H32b···*Cg*3^v^	0.98	2.75	3.6348 (17)	150

Symmetry codes: (i) −*x*+1/2, *y*−1/2, *z*; (ii) −*x*, −*y*+1, −*z*+1; (iii) −*x*+1, −*y*+1, −*z*+1; (iv) *x*, −*y*−3/2, *z*−1/2; (v) *x*−3/2, *y*, −*z*−1/2.
